# Exploring Conventional and Green Extraction Methods for Enhancing the Polyphenol Yield and Antioxidant Activity of *Hyssopus officinalis* Extracts

**DOI:** 10.3390/plants13152105

**Published:** 2024-07-29

**Authors:** Sofia Polaki, Vasiliki Stamatelopoulou, Konstantina Kotsou, Theodoros Chatzimitakos, Vassilis Athanasiadis, Eleni Bozinou, Stavros I. Lalas

**Affiliations:** Department of Food Science & Nutrition, University of Thessaly, Terma N. Temponera Str., 43100 Karditsa, Greece; spolaki@uth.gr (S.P.); vstamatel@uth.gr (V.S.); kkotsou@agr.uth.gr (K.K.); vaathanasiadis@uth.gr (V.A.); empozinou@uth.gr (E.B.); slalas@uth.gr (S.I.L.)

**Keywords:** *Hyssopus officinalis*, ultrasonication, pulsed electric field, response surface methodology, polyphenols, antioxidant activity, principal component analysis, partial least squares analysis

## Abstract

*Hyssopus officinalis* L. (HO) is, as one of the most prevalently utilized plants, used in traditional medicine to cure various diseases as well as the in food and cosmetic industries. Moreover, HO is a rich source of polyphenols with potent antioxidant properties. However, the studies on the extraction of such compounds from HO are scanty and sparse. This study aims to optimize the extraction of polyphenols and maximize the antioxidant activity in HO extracts. A comprehensive experimental design was employed, encompassing varied extraction parameters to determine the most effective ones. Alongside conventional stirring (ST), two green approaches, the ultrasonic treatment (US) and the pulsed electric field (PEF), were explored, either alone or in combination. The extracted polyphenolic compounds were identified with a high-performance liquid chromatography–diode array detector (HPLC-DAD). According to the results, the employment of ST along with an ethanolic solvent at 80 °C for 150 min seems beneficial in maximizing the extraction of polyphenols from HO, resulting in extracts with enhanced antioxidant activity. The total polyphenol was noted at 70.65 ± 2.76 mg gallic acid equivalents (GAE)/g dry weight (dw) using the aforementioned techniques, and the antioxidant activity was noted as 582.23 ± 16.88 μmol ascorbic acid equivalents (AAE)/g dw (with FRAP method) and 343.75 ± 15.61 μmol AAE/g dw (with the DPPH method). The as-prepared extracts can be utilized in the food and cosmetics industries to bestow or enhance the antioxidant properties of commercial products.

## 1. Introduction

*Hyssopus officinalis* L. (HO) is a perennial evergreen shrub of the Lamiaceae (peppermint family). It is indigenous to southern Europe and temperate regions of Asia and develops wild in countries around the Mediterranean Sea [1]. The herbs, aromatic plants, and spices of the Lamiaceae family have a high content of phenolic compounds, known for their antioxidant properties [2,3,4]. From this perspective, HO is considered among the important members of this family for their pharmaceutical potential [4] with a high profile of polyphenols [5]. At the same time, HO possesses a long history of pharmaceutical use and has been mainly used for its seasoning, tonic, antiseptic, expectorant, and analgesic properties [5,6], with HO leaves providing stimulant, stomachic, seasoning, and colic treatments, whereas the juice of the leaves is recommended for the treatment of roundworms [7]. Although slightly bitter, HO is often used in the food industry [8]; for instance, HO leaves and their flowering tops are used in salad and soup flavorings, bitters, and tonics as well as in the preparation of beverages and perfumes [1]. Notwithstanding the leaves’ therapeutic benefits, numerous studies have focused on and examined the composition of HO oil from several places worldwide [6,9,10,11,12], being further used as a flavoring agent in bitters and tonics as well as in perfumery [7].

Despite the fact that research on HO leaves is limited, there are scientific data on the total polyphenol content (TPC) in all parts of the HO plant [13,14,15,16]. More precisely, the HO essential oil received attention from Moulodi et al. [13], who isolated it through distillation. The quantification of polyphenols was completed by the Folin–Ciocalteu method, recording 23.16 mg gallic acid equivalents (GAE)/g of the extract TPC. Moreover, Fathiazad et al. [14] studied an ethyl acetate extract of the aerial part of HO after drying and defatting it. The procedure was determined spectrophotometrically according to the Folin–Ciocalteu method, exhibiting 51 mg GAE/g of TPC. HO leaf extracts were also studied spectrophotometrically by the same method. The extraction was carried out by ultrasound using ethanol, water, and 80:20% solvent. Concerning the results, 117.43 ± 9.22 mg GAE/100 g of extract (1.1743 mg GAE/g) were recorded [15]. In conclusion, even the stems of the HO plant were investigated, by Alinezhad et al. [16]. The sample was powdered and extracted with ethanol in a Soxhlet extractor. By using the aforementioned method, the TPC was recorded in great value at 374.60 ± 15.7 mg GAE/g of dry extract, ranking it as the most polyphenol-rich part of the plant.

A crucial factor for the maximization of bioactive substances, like polyphenols, constitutes the extraction technique [17]. Increasingly, traditional extraction methods, employing a simple mechanism, tend to be replaced by green and innovative extraction techniques. These new methods are believed to enhance the efficiency of the extraction and isolation of bioactive compounds, requiring less extraction time and saving solvent [17,18]. Typical examples of such methods include the ultrasound (US) [18] and pulsed electric field (PEF) techniques [19]. Exploiting the therapeutic potential and the numerous applications of HO leaves in food and cosmetics, this study undertook a systematic investigation of several extraction techniques. These included the conventional stirring (ST) [20] and the green US and PEF treatments. At the core of this research is the evaluation of the key extraction parameters such as the extraction time, extraction duration, and solvent synthesis, aiming to determine the optimal extraction procedures and optimal conditions for maximizing the TPC and antioxidant yield from HO leaves. In achieving significant improvements in the isolation of the bioactive content and antioxidant activity, the current investigation seeks to produce highly enhanced HO leaf extracts with numerous applications.

## 2. Results and Discussion

### 2.1. Extraction Optimization

For the maximization of the extraction of the TPC from HO leaves, the response surface methodology (RSM) was employed. The RSM allowed for the examination of the most significant extraction parameters, such as the extraction technique, duration, temperature, and solvent composition. For this reason, different extraction methods such as ST, US, PEF, and their combinations were tested, with the ST extraction time ranging from 15 to 150 min. As for the remaining parameters, the extraction temperature varied between 20 and 80 °C, and the solvent composition was a mixture of water and ethanol in different ratios. For the complete isolation of TPC, avoiding the successive extractions simultaneously, the used solid-to-solvent ratio was 1:20 (1 g of dried HO leaves to 20 mL of solvent).

The determination of process parameters for the US and PEF techniques is a critical step that relies on a combination of theoretical knowledge and experimental validation. Typically, parameters such as the power and frequency for the US, electric field strength, pulse period, and pulse duration for the PEF are established based on the desired outcome of the product’s physical and biochemical properties. Inappropriate parameter settings can indeed lead to unsatisfactory results, such as the suboptimal preservation of bioactive compounds or lower extraction yield. Through preliminary experiments, the optimum combination of these parameters for each extraction technique was determined, ensuring that the extraction process was efficient while maintaining the nutritional value and other parameters of the extract.

The influence of individual extraction parameters was evaluated and adjusted using the RSM method to achieve optimal extraction performance. The recorded outcomes for the 20 extracts are described in Table 1, where the variable *X*_1_ concerns the extraction technique employed, *X*_2_ concerns the concentration of ethanol in the several solvent mixtures (*C*—%, *v*/*v*), *X*_3_ refers to extraction time in min, and *X*_4_ refers to the remaining significant extraction parameter, the extraction temperature in °C. It can be seen that design points 5 and 20 were not promising, whereas, in contrast, design point 12 displayed the most encouraging results. This means that treatment with ST constitutes the optimal technique. Also, by applying an excessively high temperature (80 °C) for a 150 min extraction duration using ethanolic solvent (25%), a highly enriched extract can be obtained. In our study, it appears that the RSM has successfully illustrated the consistency between actual and predicted values across a range of design points.

**Figure 1:**
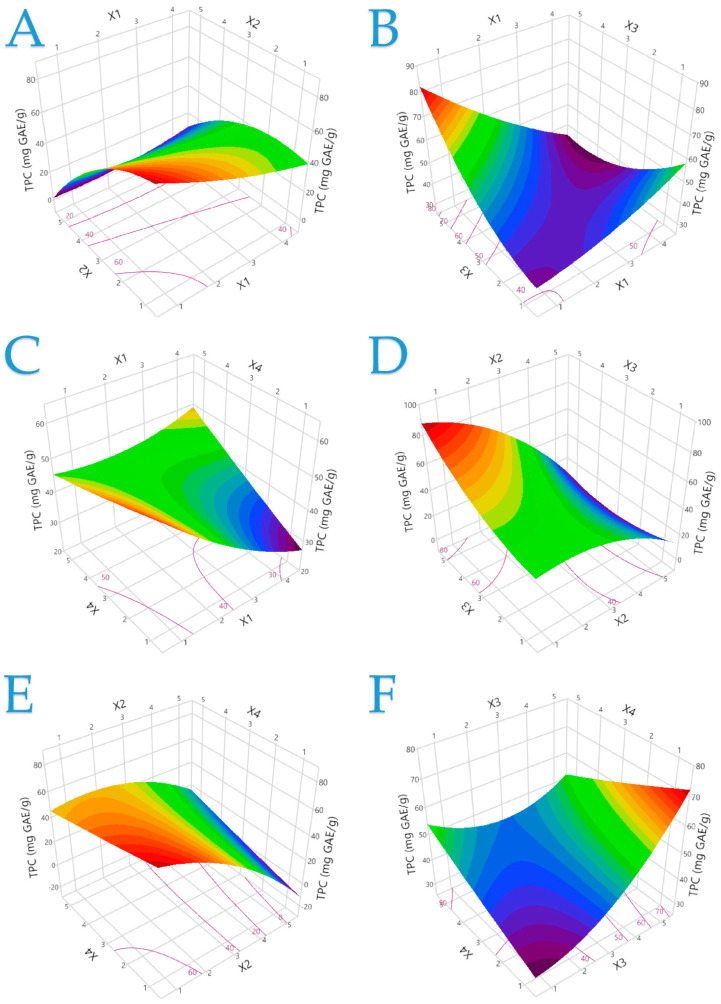
The optimal extraction of the HO plant is shown in 3D graphs that show the impact of the process variables considered in the response (total polyphenol content—TPC, mg GAE/g). Plot (**A**), covariation of *X*_1_ and *X*_2_; plot (**B**), covariation of *X*_1_ and *X*_3_; plot (**C**), covariation of *X*_1_ and *X*_4_; plot (**D**), covariation of *X*_2_ and *X*_3_; plot (**E**), covariation of *X*_2_ and *X*_4_; plot (**F**), covariation of *X*_3_ and *X*_4_.

Figures S1–S3 display plots showing a comparison of the actual response with the predicted response for each parameter analyzed, including the desirability functions. Moreover, responses in three-dimensional plots are presented in Figure 1, Figures S4 and S5, providing a clearer view of the dependence of the TPC on the parameters under study. Similar three-dimensional response plots (Figures S4 and S5) for the parameters of antioxidant activity (FRAP and DPPH) can be found in the Supplementary Materials. Table 2 lists all statistical parameters, second-order polynomial equations (models), and coefficients (with values greater than 0.98) for each model. An indication of a strong correlation between the established models and the data is the high coefficient values, ideally 1.

### 2.2. Impact of Extraction Parameters on Assays through Pareto Plot Analysis

**Figure 2:**
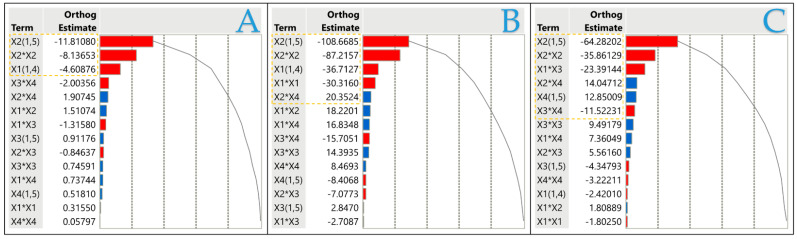
Pareto plots of transformed estimates for TPC (**A**), FRAP (**B**), and DPPH (**C**) assays. A rectangular gold reference line is drawn on the plot to indicate the significance level (*p* < 0.05).

In Figure 2, the relevance of each extraction factor to the efficiency of bioactive component isolation is strongly illustrated, showing both positive and negative correlations. In particular, a negative association with the solvent concentration is discernible with respect to both the TPC and antioxidant capacity. Contrary to conventional expectations, neither pure water nor high concentrations of ethanol emerge as optimal solvents for the extraction of the maximum amounts of bioactive compounds. Instead, an excellent balance is found within a certain concentration of ethanol, 25%.

The findings highlighted in Figure 2 strongly coordinate with the comprehensive analyses presented in Table 1. In particular, the highest levels of bioactive compounds and antioxidant activity are precisely achieved using the 25% ethanol solvent extraction method. This revelation highlights the value of solvent selection, emphasizing the critical importance of precision and optimization in extraction methods to isolate the full potential of bioactive compounds present in natural resources.

### 2.3. Analysis of the Extracts

#### 2.3.1. TPC of the Extracts

The method used for the quantification of TPC, despite its limitations, was the Folin–Ciocalteu method. The limitations lie in the fact that the reagent reacts positively with amino acids as well as several organic and inorganic substances [21]. Nevertheless, it is the most commonly used method by scientists for the determination of total phenolic compounds [21]. Examining Table 1, the TPC in HO leaves occurred between 5.61 and 66.61 mg GAE/g dw, noting an enhancement of 1087.34%. The maximum amount was observed after the ST treatment at 80 °C for 150 min. Whereas many polyphenols are thermosensitive compounds, extractions at temperatures above 80 °C often provide impaired efficiencies [22,23], and the present outcome suggests the fact that the contained polyphenols are less thermosensitive. As reported above, in a previous survey, the TPC in HO leaves was 1.17 mg GAE/g [15], i.e., 379.49–5593.16% less than the minimum and maximum amount recorded in this study. Regarding the aerial part from the HO plant, the TPC did not exceed 51 mg GAE/g [14], a value 30.61% less than the value that can be completed in leaves applying the proposed conditions. These results emphasize the importance and value of selecting all the parameters that can influence an extraction in order to achieve the optimal result and create a highly bio-functional extract.

For all measured parameters, Table 3 presents the responses of these parameters obtained using the optimal conditions derived through the least squares method. The conventional ST extraction technique was highlighted as the most suitable for all analyses, together with an extraction time of 150 min. The conventional ST extraction technique was highlighted as the most suitable for all analyses, together with an extraction time of 150 min. In previous studies, the use of green extraction methods benefited the total polyphenols profile of the extracts [19,24,25], although, in terms of HO leaves, the US or PEF pretreatments were not considered necessary to ensure optimal values, according to Table 1 and Table 3. This result may be attributed to the fact that green techniques themselves have certain drawbacks. Specifically, the US technique encounters challenges in scaling up and diminished efficiency in systems with high viscosity, such as ethanol, and non-selective extraction [26]. Moreover, treatment with the PEF not only faces scaling-up challenges but also poses a risk of compound oxidation [26]. Furthermore, the hydroethanolic mixture (75% deionized water and 25% ethanol) was found to be the most appropriate extraction solvent for all investigated parameters. In general, it is reported that ethanol (either as such or in an aqueous mixture) is recommended for the extraction of polyphenolic compounds from plant sources [27]. This statement is scientifically correct, as polyphenols are characterized by low polarity, which requires the presence of a low-polarity solvent (such as ethanol) for their adequate isolation [28]. Moreover, ethanol is an easily recoverable solvent, using a rotary evaporator, allowing its reuse, while being considered a suitable solvent for human consumption, and is, therefore, widely chosen by the food and pharmaceutical industries [27,28]. These were the main reasons for the selection of ethanol (in various proportions) as an extraction solvent. Table 4 illustrates the predicted values (according to the PLS analysis) compared to the experimental values recorded. Considering the standard deviations, these values are fully comparable to the predicted values. Upon comparing the PLS model’s values with those obtained after experimental analysis, the correlation among them is found to be 0.9976, and they show no deviations, with the *p*-value being <0.0001. According to our study, the experimental and predicted values of the experiments are appropriately reflected in a well-fitting model.

#### 2.3.2. Antioxidant Properties of the Extracts

In order to ensure an in-depth study of the antioxidant activity of the different HO extract samples, two methods that evaluate the antioxidant activity, the iron reduction antioxidant power (FRAP) and the free radical scavenging antioxidant capacity (DPPH) were tested. Although these analyses are widely used and reliable, they also present limitations in their applications [29,30]. More specifically, in terms of the FRAP, for example, the most important limitation is the redox potential of the Fe^3+^/Fe^2+^ pair. Any compound with a redox potential lower than this may produce falsely high Fe^3+^ reduction results; thus, higher antioxidant capacity results [30], and simultaneously, the DPPH, is technically simple, but it is a long-lived nitrogen radical, and many antioxidants that react quickly with other radicals, such as peroxyl radical, may react slowly or even be inert to the DPPH [29]. According to Table 1 and Table 3, it can be seen that the maximum antioxidant capacity value, regardless of the method, is obtained by applying the same extraction conditions, which perfectly coincide with the optimal conditions of the maximum TPC. In particular, after treatment with simple ST, at the maximum temperature (80 °C) and time (150 min), and 25% ethanolic solvent, the antioxidant capacity can be increased up to 882.02% with the FRAP method and 805.46% with the DPPH method. Antioxidant activity is an outstanding instance of the functional benefits that plant extracts can provide, which has been significantly recognized and appreciated by the modern world [31,32]. For example, in terms of the cosmetics industry, in addition to the reduction in oxidation having a clear benefit for cosmetic products, antioxidants are highly attractive and preferred as cosmetic ingredients [32]. In view of the fact that both the leaves and flowers of the HO plant are already used for the preparation of cosmetic products [33], the resulting extract could be easily prepared by the cosmetic industry and used as a strong natural antioxidant extract in various products. Last but not least, natural antioxidants, which are mainly derived from plant materials such as herbs [31], have the ability to inhibit the growth of microorganisms such as *Salmonella* spp. and *Escherichia coli* [34] and are, therefore, often used as ingredients in various food products [31]. This fact gives a strong impetus to the use of HO leaf extract also in the food sector [35].

#### 2.3.3. Polyphenolic Compounds of the Optimal Extract

For the identification and quantification of the polyphenolic elements, all available equipment was used, but it is important to mention that some limitations were presented for the identification of all the polyphenolic elements contained in the HO leaves. Nevertheless, the spectra showed 100% purity compared to the standards and no co-elution with other elements that occurred in any of the spectra. However, a plethora of polyphenolic compounds, in significant amounts, were identified in the optimum extract of HO leaves, as shown in Table 5. A representative chromatogram can be seen in Figure S6, while in Table S1, data regarding the identification and quantification of the compounds are presented. In previous surveys, where the polyphenolic compounds contained in the HO plant have been examined, chlorogenic [4,36,37], caffeic [4,36,37,38], ferulic [36,37,38], syringic [36,37], and *p*-coumaric [4,37] acids were highlighted. Moreover, rutin [4,37], quercetin [4,36,37], apigenin [36], apigenin 7-*O*-β-D-glucuronide [37], luteolin [4,36,37], and catechin [11] were recorded. Based on the findings of these studies, there is a perfect match with the results of the present study, as shown in Table 5, except that significant amounts of neochlorogenic acid and small amounts of vanillic acid were identified in the present study. In descending order, the leading elements appear to be apigenin-7-*O*-glucoside, rutin, *p*-coumaric acid, and caffeic acid, constituting 17.9, 16.9, 14.11, and 13.37% of the total amount of the polyphenolic compounds identified and quantified. Rutin and *p*-coumaric acid are known for their medicinal properties, are considered suitable for the treatment of cardiovascular diseases [39,40], and are widely reputed for their antioxidant, antimicrobial, anti-inflammatory, and anticancer properties [40,41]. As is prevalent in the optimal sample, these polyphenolic compounds impart these properties to the sample as well, enhancing its therapeutic properties. In addition, caffeic acid is also renowned for its antioxidant properties, preventing oxidative stress [42]. Srivastava et al. [38], who studied the HO plant collected in India, recorded values of 0.146 mg/g for caffeic acid, values which are negligible compared to the amount that can be obtained by applying the proposed extraction conditions to the HO plant. In the same study, chlorogenic acid was recorded at 1.20 mg/g, 228.33% less than the amount secured in the present study, without it being one of the main polyphenolic compounds. It is, therefore, concluded that the extract of HO leaves can be rendered highly beneficial and enhanced by applying the optimum extraction conditions.

### 2.4. Principal Component Analysis (PCA) and Multivariate Correlation Analysis (MCA)

**Figure 3:**
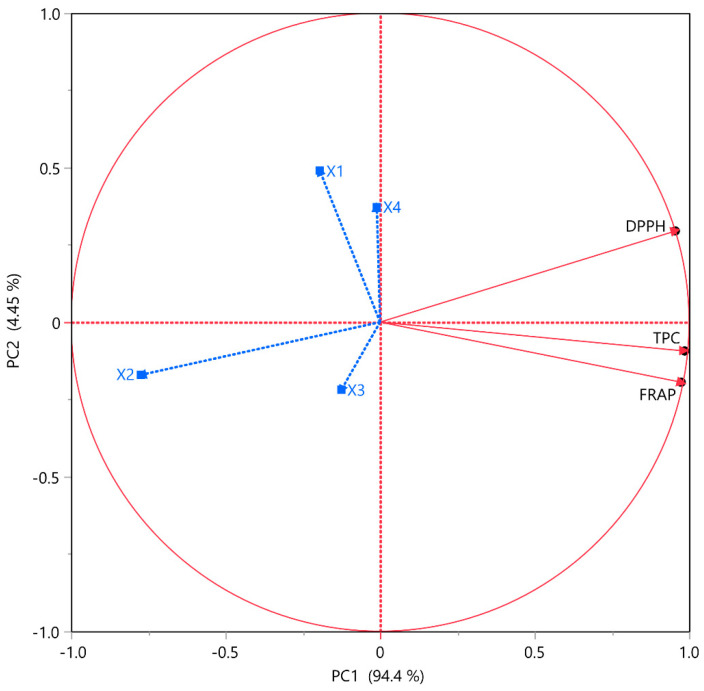
Principal component analysis (PCA) for the measured variables. Each *X* variable is presented with a blue color.

Principal component analysis (PCA) represents a crucial statistical technique as it enables the simplification of complex data sets while preserving their fundamental characteristics. PCA is a valuable tool for exploratory analysis and data visualization. Three PCA technical replicates were performed, ensuring the validity and accuracy of the results. The results were found identical in all replicates. Figure 3 and Table 6 illustrate the correlation of values between different bioactive compounds, revealing appreciable results. PC1, indicated in Figure 3, describes 94.4% of the variability, with the different tested variables TPC, FRAP, and DPPH in HO leaf extract recording positive correlation. Among the extraction factors, the one that appears to have the greatest influence on the extraction results is *X*_2_, as demonstrated in Figure 2, with *X*_1_ following but without exhibiting such a strong influence. It is worth mentioning again that the optimum solvent mixture was the aqueous ethanolic mixture (75:25).

With a peak correlation value of 1, Table 6 presents the correlation between the three variables examined, displaying an excellent correlation. Delving further, the TPC shows 0.96 and 0.91 between the antioxidant actions FRAP and DPPH, respectively. Hence, it is inferred that the abundance of polyphenols and polyphenolic compounds confers a rich antioxidant capacity along with intense free radical scavenging properties to the HO extract. Moreover, as mentioned above, all the polyphenolic compounds identified are known for their antioxidant capacities. Free radicals are frequently attributed to oxidative stress and cancer occurrence [43]; therefore, an assumption might be that the polyphenols of the HO sample exhibit anticancer properties, as various polyphenols are reported to possess [44,45,46], due to their free radical binding capacity.

### 2.5. Partial Least Squares (PLS) Analysis

**Figure 4:**
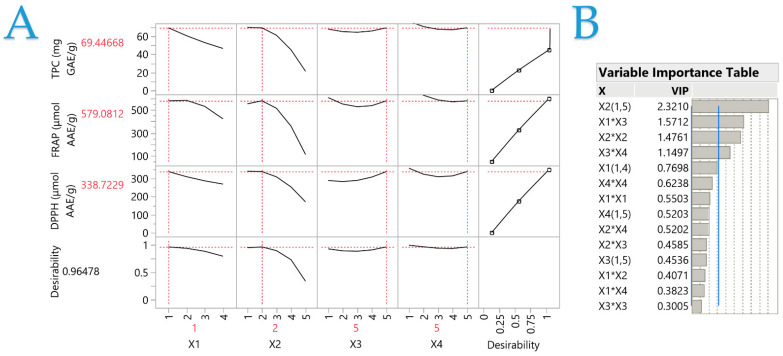
Plot (**A**) displays the desirability function and partial least squares (PLS) prediction profiler and desirability function with extrapolation control for optimizing *H. officinalis* plant. Plot (**B**) table displays the VIP values for each predictor variable on the Variable Importance Plot (VIP) option graph. The VIT at 0.8 shows a blue dashed line representing each variable’s significance level.

The impact of various extraction parameters (*X*_1_, *X*_2_, *X*_3_, and *X*_4_) was evaluated through a partial least squares (PLS) model, as illustrated in the correlation loading plot depicted in Figure 4A. In this plot, the influence of extraction conditions on HO leaf extracts is elucidated. It was determined that the *X*_2_ variable exerted the most significant effect on optimizing responses across all assays, as evidenced in Figure 2 and Figure 3. It is evident that an increase in ethanol concentration results in a decrease in extraction efficiency, with a plateau observed at 25% ethanol. Concerning *X*_1_, the implementation of extraction pretreatments appears to diminish extraction efficiency, particularly in the TPC and DPPH assays. In the case of the FRAP method, the PEF pretreatment was identified as a suitable extraction technique, though it did not achieve the maximal results seen with the ST technique.

As depicted in Figure 4B, the significance of each extraction parameter concerning TPC levels and antioxidant activity is emphasized. *X*_2_, representing the solvent composition, emerges as the predominant and most pivotal factor, with an influence value approaching 2.3, significantly surpassing the critical threshold of 0.8 for each variable. Furthermore, the interactions between the extraction technique and extraction duration, as well as between the extraction duration and temperature, exhibit considerable influence, with impact values exceeding 1. These results underscore the essential role of the solvent concentration in the efficiency of the extraction process and the creation of the most potent extract.

## 3. Materials and Methods

### 3.1. Chemicals and Reagents

All chemicals and reagents are presented in detail in the Supplementary Materials.

### 3.2. Extraction Procedure

HO leaves were sourced from a local store in the Karditsa Region of Greece. The samples were then placed in a Biobase BK-FD10P lyophilizer (Jinan, China), where they underwent a 12 h moisture removal process. After drying, the leaves were ground into a fine powder and subsequently stored at −40 °C until further examination.

A total of 1 g of HO powder was mixed with 20 mL of extraction solvent. This ratio was optimized in preliminary experiments for maximum total polyphenol extraction. Specific extraction parameters can be found in Table 7. The samples were stirred at 500 rpm under various temperature and time conditions. Before the ST extraction, some samples were treated with additional green extraction methods. One such method was PEF, which involved a pulse period of 1 ms (frequency: 1 kHz), a pulse duration of 10 μs, and an electric field strength of 1.0 kV/cm. This setup included a mode/arbitrary waveform generator (UPG100, ELV Elektronik AG, Leer, Germany), a digital oscilloscope (Rigol DS1052E, Beaverton, OR, USA), a high-voltage power generator (Leybold, LD Didactic GmbH, Huerth, Germany), and custom stainless-steel chambers (Val-Electronic, Athens, Greece). Additionally, US was the second additional extraction technique, which was performed using an Elmasonic P machine, 180 W (Elma Schmidbauer GmbH, Singen, Germany), at 30 °C and 37 kHz. The dried material was pre-soaked in the solvent for 10 min before any treatment. After extraction, the mixture was centrifuged at 4500 rpm for 10 min, and the supernatant was collected for the aforementioned analyses.

### 3.3. Response Surface Methodology (RSM) Extraction Optimization and Design of Experiment

In the Supplementary Materials, extensive information on the application of response surface methodology (RSM) for improvement of the efficiency of the extraction procedure of the HO sample is included.

### 3.4. Analysis of the Extracts

The dry weight (dw) of the HO plant was used to express the results. The total polyphenol content (TPC) of HO extracts was calculated using the procedure outlined by Kotsou et al. [47]. Using the Ferric Reducing Antioxidant Power (FRAP) assay, the antioxidant activity was assessed in accordance with the previously published protocol by Chatzimitakos et al. [48]. The methodology for evaluating the DPPH radical scavenging activity was conducted according to the earlier description provided by Chatzimitakos et al. [48].

### 3.5. HPLC-Based Analysis of the Polyphenolic Compounds

High-performance liquid chromatography (HPLC) was employed to quantify individual polyphenolic compounds, as was noted in our earlier study [49]. The HO extracts were analyzed using an HPLC-DAD system (Shimadzu Europa GmbH, Duisburg, Germany), and the compounds were divided into a Phenomenex Luna C18(2) column from Phenomenex Inc., Torrance, CA, USA (100 Å, 5 µm, 4.6 mm × 250 mm) at a temperature of 40 °C. The mobile phase consisted of 0.5% formic acid in acetonitrile (B) and 0.5% formic acid in aqueous solution (A). First, from 0 to 40% B, followed by 50% B in 10 min, 70% B in another 10 min, and then constant for 10 min was the gradient program that was needed. The flow rate of the mobile phase was 1 mL/min. The identification process involved comparing the absorbance spectrum and retention time to those of pure standards, followed by quantification using calibration curves ranging from 0 to 50 μg/mL.

### 3.6. Statistical Analysis

Response surface methodology and distribution analysis were the subjects of the statistical analysis that was performed using JMP^®^ Pro 16 software (SAS, Cary, NC, USA). The extraction methods were carried out at least twice for every batch of HO extract, and the quantitative analysis was carried out in triplicate. The means and standard deviations of the results were used to illustrate them. The JMP^®^ Pro 16 software was used to perform principal component analysis (PCA), multivariate correlation analysis (MCA), and partial least squares (PLS) analysis.

## 4. Conclusions

The findings of this study shed light on the extraction optimization/modeling of HO leaves and reveal their diverse bioactive compounds and antioxidant properties. Using a meticulously designed experimental design, this study revealed the most effective method: the use of conventional stirring (ST) with an ethanolic solvent at 80 °C for 150 min. This strategic approach demonstrated that an extract of HO can be prepared that is a remarkable source of polyphenols, with strong antioxidant properties that can be exploited in various fields. Going deeper, analysis of HO leaves revealed a rich array of polyphenolic compounds, including rutin, *p*-coumaric acid, and caffeic acid. These compounds, known for their medicinal properties and antioxidant potency, further underscore the therapeutic promise contained in HO extract. In essence, this study not only presents the most suitable extraction parameters but also illuminates the intrinsic properties of HO, and it also points toward its vast potential across industries. From the fortification of pharmaceutical formulations to the enrichment of food products and cosmetic preparations, the versatile utility of the HO extract will play a key role in enhancing the efficacy of a plethora of products. To conclude, this research would serve as an excellent basis to examine and evaluate the estimated actions of the HO leaves.

## Figures and Tables

**Table 1:** Experimental findings for the four independent variables under investigation and the dependent variable’s responses.

Design Point	Independent Variables				Responses					
					TPC (mg GAE/g dw)		FRAP (μmol AAE/g dw)		DPPH (μmol AAE/g dw)	
	*X*_1_	*X*_2_	*X*_3_	*X*_4_	Actual	Predicted	Actual	Predicted	Actual	Predicted
1	3	1	3	4	46.52 ± 1.21	46.88	432.73 ± 31.16	402.89	304.30 ± 21.91	292.75
2	3	2	1	3	46.65 ± 1.49	47.97	486.93 ± 16.56	490.81	295.66 ± 12.42	294.00
3	2	3	4	3	45.03 ± 3.11	48.36	450.67 ± 26.14	467.22	235.21 ± 10.35	224.37
4	2	4	5	4	43.40 ± 1.52	38.02	383.74 ± 16.51	363.67	216.25 ± 16.03	219.97
5	3	5	4	2	7.33 ± 0.28	6.43	63.60 ± 1.78	69.88	37.20 ± 2.38	36.67
6	4	1	4	5	41.42 ± 2.98	40.57	317.26 ± 17.45	327.95	260.82 ± 8.35	266.85
7	4	2	3	1	41.47 ± 2.53	40.36	338.64 ± 23.72	325.38	227.57 ± 9.79	221.13
8	1	3	3	2	51.53 ± 1.91	50.11	464.65 ± 17.19	439.07	214.76 ± 13.15	204.81
9	1	4	4	1	40.82 ± 1.39	41.83	354.61 ± 24.82	355.58	217.91 ± 7.41	217.93
10	1	5	1	4	17.08 ± 0.41	17.44	110.37 ± 8.06	108.50	74.11 ± 4.22	71.13
11	1	1	2	3	60.16 ± 3.31	57.43	459.62 ± 19.76	466.33	254.15 ± 12.45	262.16
12	1	2	5	5	66.61 ± 3.53	68.76	567.61 ± 12.49	573.07	336.83 ± 9.09	334.81
13	4	3	2	4	45.73 ± 2.42	47.56	387.63 ± 18.99	416.43	287.68 ± 8.92	297.61
14	3	4	2	5	45.37 ± 3.36	42.87	484.76 ± 11.15	449.73	250.55 ± 13.53	234.62
15	2	5	3	5	18.98 ± 0.42	20.91	139.97 ± 7.42	167.05	130.68 ± 3.92	144.73
16	2	1	1	1	49.64 ± 2.58	50.52	519.93 ± 22.88	518.83	310.48 ± 14.9	302.46
17	2	2	2	2	47.77 ± 3.11	48.89	500.31 ± 24.01	500.81	201.38 ± 10.07	223.22
18	3	3	5	1	49.76 ± 2.04	49.60	514.24 ± 19.03	525.99	211.91 ± 13.77	220.83
19	4	4	1	2	36.33 ± 2.33	34.95	312.37 ± 13.74	307.90	233.40 ± 7.94	236.45
20	4	5	5	3	5.61 ± 0.13	7.74	57.80 ± 2.14	50.35	50.34 ± 1.51	44.69

**Table 2:** Mathematical models developed with RSM were employed to maximize the extraction from the HO plant. Only important terms were present in the models.

Responses	Second-Order Polynomial Equations (Models)	R^2^ Predicted	R^2^ Adjusted	*p*-Value	Equation
TPC	*Y* = 63.7 − 9.38*X*_1_ − 3.79*X*_2_ + 12.4*X*_3_ − 5.52*X*_4_ + 0.8*X*_1_^2^ − 2.18*X*_2_^2^ + 1.41*X*_3_^2^ + 0.07*X*_4_^2^ + 2.2*X*_1_*X*_2_ − 3.31*X*_1_*X*_3_ + 1.76*X*_1_*X*_4_ − 1.84*X*_2_*X*_3_ + 2.2*X*_2_*X*_4_ − 1.67*X*_3_*X*_4_	0.9834	0.9368	0.0017	(1)
FRAP	*Y* = 552.36 + 24.52*X*_1_ + 79.2*X*_2_ + 17.83*X*_3_ − 102.06*X*_4_ − 28.02*X*_1_^2^ − 38.14*X*_2_^2^ + 17.88*X*_3_^2^ + 9.24*X*_4_^2^ + 25.73*X*_1_*X*_2_ − 16.14*X*_1_*X*_3_ + 19.55*X*_1_*X*_4_ − 14.29*X*_2_*X*_3_ + 13.52*X*_2_*X*_4_ − 11.97*X*_3_*X*_4_	0.9884	0.9560	0.0007	(2)
DPPH	*Y* = 332.45 + 26.97*X*_1_ − 138.46*X*_2_ + 90.18*X*_3_ − 41.75*X*_4_ + 7.26*X*_1_^2^ + 6.67*X*_2_^2^ + 13.38*X*_3_^2^ − 3.83*X*_4_^2^ + 2.39*X*_1_*X*_2_ − 39.62*X*_1_*X*_3_ + 17.34*X*_1_*X*_4_ − 8.49*X*_2_*X*_3_ + 19.93*X*_2_*X*_4_ − 10.82*X*_3_*X*_4_	0.9872	0.9513	0.0009	(3)

**Table 3:** Maximum predicted responses and optimum extraction conditions for the dependent variables.

Responses	Optimal Conditions				
	Maximum Predicted Response	Technique (*X*_1_)	*C* (%, *v*/*v*) (*X*_2_)	*t* (min) (*X*_3_)	*T* (°C) (*X*_4_)
TPC (mg GAE/g dw)	68.76 ± 9.90	ST (1)	25 (2)	150 (5)	80 (5)
FRAP (μmol AAE/g dw)	572.44 ± 81.06	ST (1)	25 (2)	150 (5)	80 (5)
DPPH (μmol AAE/g dw)	334.81 ± 45.62	ST (1)	25 (2)	150 (5)	80 (5)

**Table 4:** Maximum desirability for all variables using the partial least squares (PLS) prediction profiler under the optimal extraction conditions (*X*_1_:1, *X*_2_:2, *X*_3_:5, and *X*_4_:5).

Variables	PLS Model Values	Experimental Values
TPC (mg GAE/g dw)	69.45	70.65 ± 2.76
FRAP (μmol AAE/g dw)	579.08	582.23 ± 16.88
DPPH (μmol AAE/g dw)	338.72	343.75 ± 15.61

**Table 5:** Polyphenolic compounds under optimal extraction conditions (*X*_1_:1, *X*_2_:2, *X*_3_:5, and *X*_4_:5).

Polyphenolic Compound	Optimal Extract (mg/g dw)
Neochlorogenic acid	1.26 ± 0.04
Catechin	2.8 ± 0.1
Chlorogenic acid	3.94 ± 0.1
Vanillic acid	0.13 ± 0
Caffeic acid	5.55 ± 0.12
Syringic acid	3.14 ± 0.15
*p*-Coumaric acid	6.89 ± 0.39
Ferulic acid	4.57 ± 0.16
Rutin	8.25 ± 0.57
Quercetin 3-*D*-galactoside	0.4 ± 0.01
Luteolin-7-glucoside	1.79 ± 0.09
Apigenin-7-*O*-glucoside	8.74 ± 0.36
Apigenin	1.37 ± 0.03
Total identified	48.83 ± 2.11

**Table 6:** Multivariate correlation analysis of measured variables.

Responses	TPC	FRAP	DPPH
TPC	−	0.9624	0.9095
FRAP		−	0.8767
DPPH			−

**Table 7:** The actual and coded levels of the independent variables were used to optimize the process.

Independent Variables	Code Units	Coded Variable Level				
		1	2	3	4	5
Technique	*X*_1_	ST	PEF + ST	US + ST	PEF + US + ST	–
*C* (%, *v*/*v*)	*X*_2_	0	25	50	75	100
*t* (min)	*X*_3_	30	60	90	120	150
*T* (°C)	*X*_4_	20	35	50	65	80

## Data Availability

All related data and methods are presented in this paper. Additional inquiries should be addressed to the corresponding author.

## Supplementary Materials

The following supporting information can be downloaded at: https://www.mdpi.com/article/10.3390/plants13152105/s1, Figures S1–S3 comprise plots that illustrate the comparison between the actual response and the predicted response for each parameter under examination, accompanied by the desirability functions. Figures S4 and S5 present three-dimensional response plots for the remaining responses. Figure S6 shows an HPLC chromatogram at 320 nm of the optimal extract. Table S1 presents data for the identification and quantification of polyphenolic compounds in the extracts.

## References

[1] Ravindran P.N., Divakaran M., Pillai G.S., Peter K.V. (2012). Other Herbs and Spices: Achiote to Szechuan Pepper. Handbook of Herbs and Spices.

[2] Ciocârlan V. (2009). Illustrated Flora of Romania. Pteridophyta et Spermatophyta.

[3] Lobo V., Patil A., Phatak A., Chandra N. (2010). Free Radicals, Antioxidants and Functional Foods: Impact on Human Health. Pharmacogn. Rev..

[4] Vlase L., Benedec D., Hanganu D., Damian G., Csillag I., Sevastre B., Mot A.C., Silaghi-Dumitrescu R., Tilea I. (2014). Evaluation of Antioxidant and Antimicrobial Activities and Phenolic Profile for *Hyssopus officinalis*, *Ocimum basilicum* and *Teucrium chamaedrys*. Molecules.

[5] Kazazi H., Rezaei K., Ghotbsharif S., Emamdjomeh Z., Yamini Y. (2007). Supercriticial Fluid Extraction of Flavors and Fragrances from *Hyssopus officinalis* L. Cultivated in Iran. Food Chem..

[6] Khazaie H.R., Nadjafi F., Bannayan M. (2008). Effect of Irrigation Frequency and Planting Density on Herbage Biomass and Oil Production of Thyme (*Thymus vulgaris*) and Hyssop (*Hyssopus officinalis*). Ind. Crops Prod..

[7] Ravindran P.N., Pillai G.S., Nirmal Babu K., Peter K.V. (2004). Under-Utilized Herbs and Spices. Handbook of Herbs and Spices.

[8] Judžentienė A., Preedy V.R. (2016). Hyssop (*Hyssopus officinalis* L.) oils. Essential Oils in Food Preservation, Flavor and Safety.

[9] Langa E., Cacho J., Palavra A.M.F., Burillo J., Mainar A.M., Urieta J.S. (2009). The Evolution of Hyssop Oil Composition in the Supercritical Extraction Curve. J. Supercrit. Fluids.

[10] Fraternale D., Ricci D., Epifano F., Curini M. (2004). Composition and Antifungal Activity of Two Essential Oils of Hyssop (*Hyssopus officinalis* L.). J. Essent. Oil Res..

[11] Džamić A.M., Soković M.D., Novaković M., Jadranin M., Ristić M.S., Tešević V., Marin P.D. (2013). Composition, Antifungal and Antioxidant Properties of *Hyssopus officinalis* L. Subsp. Pilifer (Pant.) Murb. Essential Oil and Deodorized Extracts. Ind. Crops Prod..

[12] Zawislak G. (2016). Essential Oil Composition of *Hyssopus officinalis* L. Grown in Poland. J. Essent. Oil-Bear. Plants.

[13] Moulodi F., Khezerlou A., Zolfaghari H., Mohamadzadeh A., Alimoradi F. (2019). Chemical Composition and Antioxidant and Antimicrobial Properties of the Essential Oil of *Hyssopus officinalis* L.. J. Kermanshah Univ. Med. Sci..

[14] Fathiazad F., Mazandarani M., Hamedeyazdan S. (2011). Phytochemical Analysis and Antioxidant Activity of *Hyssopus officinalis* L. from Iran. Adv. Pharm. Bull..

[15] Rezaei Savadkouhi N., Ariaii P., Charmchian Langerodi M. (2020). The Effect of Encapsulated Plant Extract of Hyssop (*Hyssopus officinalis* L.) in Biopolymer Nanoemulsions of *Lepidium perfoliatum* and *Orchis mascula* on Controlling Oxidative Stability of Soybean Oil. Food Sci. Nutr..

[16] Alinezhad H., Azimi R., Zare M., Ebrahimzadeh M.A., Eslami S., Nabavi S.F., Nabavi S.M. (2013). Antioxidant and Antihemolytic Activities of Ethanolic Extract of Flowers, Leaves, and Stems of *Hyssopus officinalis* L. Var. *angustifolius*. Int. J. Food Prop..

[17] Panja P. (2018). Green Extraction Methods of Food Polyphenols from Vegetable Materials. Curr. Opin. Food Sci..

[18] Koina I.M., Sarigiannis Y., Hapeshi E. (2023). Green Extraction Techniques for the Determination of Active Ingredients in Tea: Current State, Challenges, and Future Perspectives. Separations.

[19] Athanasiadis V., Chatzimitakos T., Kotsou K., Kalompatsios D., Bozinou E., Lalas S.I. (2023). Polyphenol Extraction from Food (by) Products by Pulsed Electric Field: A Review. Int. J. Mol. Sci..

[20] Gharaati Jahromi S., Soto-Hernández M., García-Mateos R., Palma-Tenango M. (2019). Extraction Techniques of Phenolic Compounds from Plants. Plant Physiological Aspects of Phenolic Compounds.

[21] Górnaś P., Dwiecki K., Siger A., Tomaszewska-Gras J., Michalak M., Polewski K. (2016). Contribution of Phenolic Acids Isolated from Green and Roasted Boiled-Type Coffee Brews to Total Coffee Antioxidant Capacity. Eur. Food Res. Technol..

[22] Lang G.H., da Silva Lindemann I., Ferreira C.D., Hoffmann J.F., Vanier N.L., de Oliveira M. (2019). Effects of Drying Temperature and Long-Term Storage Conditions on Black Rice Phenolic Compounds. Food Chem..

[23] Antony A., Farid M. (2022). Effect of Temperatures on Polyphenols during Extraction. Appl. Sci..

[24] Diamanti A.C., Igoumenidis P.E., Mourtzinos I., Yannakopoulou K., Karathanos V.T. (2017). Green Extraction of Polyphenols from Whole Pomegranate Fruit Using Cyclodextrins. Food Chem..

[25] Zannou O., Pashazadeh H., Ghellam M., Ali Redha A., Koca I. (2022). Enhanced Ultrasonically Assisted Extraction of Bitter Melon (*Momordica charantia*) Leaf Phenolic Compounds Using Choline Chloride-Acetic Acid–Based Natural Deep Eutectic Solvent: An Optimization Approach and in Vitro Digestion. Biomass Conv. Biorefin..

[26] Calderón-Oliver M., Ponce-Alquicira E. (2021). Environmentally Friendly Techniques and Their Comparison in the Extraction of Natural Antioxidants from Green Tea, Rosemary, Clove, and Oregano. Molecules.

[27] Hikmawanti N.P.E., Fatmawati S., Asri A.W. (2021). The Effect of Ethanol Concentrations as the Extraction Solvent on Antioxidant Activity of Katuk (*Sauropus androgynus* (L.) Merr.) Leaves Extracts. IOP Conf. Ser. Earth Environ. Sci..

[28] Nguyen N.Q., Nguyen M.T., Nguyen V.T., Le V.M., Trieu L.H., Le X.T., Khang T.V., Giang N.T.L., Thach N.Q., Hung T.T. (2020). The Effects of Different Extraction Conditions on the Polyphenol, Flavonoids Components and Antioxidant Activity of *Polyscias fruticosa* Roots. IOP Conf. Ser. Mater. Sci. Eng..

[29] Huang D., Ou B., Prior R.L. (2005). The Chemistry behind Antioxidant Capacity Assays. J. Agric. Food Chem..

[30] Wojtunik-Kulesza K.A. (2020). Approach to Optimization of FRAP Methodology for Studies Based on Selected Monoterpenes. Molecules.

[31] Flieger J., Flieger W., Baj J., Maciejewski R. (2021). Antioxidants: Classification, Natural Sources, Activity/Capacity Measurements, and Usefulness for the Synthesis of Nanoparticles. Materials.

[32] Angerhofer C.K., Maes D., Giacomoni P.U., Dayan N. (2009). The Use of Natural Compounds and Botanicals in the Development of Anti-Aging Skin Care Products. Skin Aging Handbook.

[33] Sharifi-Rad J., Quispe C., Kumar M., Akram M., Amin M., Iqbal M., Koirala N., Sytar O., Kregiel D., Nicola S. (2022). Hyssopus Essential Oil: An Update of Its Phytochemistry, Biological Activities, and Safety Profile. Oxid. Med. Cell. Longev..

[34] Cetin-Karaca H., Newman M.C. (2015). Antimicrobial Efficacy of Plant Phenolic Compounds against Salmonella and *Escherichia coli*. Food Biosci..

[35] Caleja C., Barros L., Antonio A.L., Ciric A., Barreira J.C.M., Sokovic M., Oliveira M.B.P.P., Santos-Buelga C., Ferreira I.C.F.R. (2015). Development of a Functional Dairy Food: Exploring Bioactive and Preservation Effects of Chamomile (*Matricaria recutita* L.). J. Funct. Foods.

[36] Fathiazad F., Hamedeyazdan S. (2011). A Review on *Hyssopus officinalis* L: Composition and Biological Activities. Afr. J. Pharm. Pharmacol..

[37] Skrypnik L., Feduraev P., Styran T., Golovin A., Katserov D., Nebreeva S., Maslennikov P. (2022). Biomass, Phenolic Compounds, Essential Oil Content, and Antioxidant Properties of Hyssop (*Hyssopus officinalis* L.) Grown in Hydroponics as Affected by Treatment Type and Selenium Concentration. Horticulturae.

[38] Srivastava A., Awasthi K., Kumar B., Misra A., Srivastava S. (2018). Pharmacognostic and Pharmacological Evaluation of *Hyssopus officinalis* L. (Lamiaceae) Collected from Kashmir Himalayas, India. Pharmacogn. J..

[39] Dolrahman N., Mukkhaphrom W., Sutirek J., Thong-Asa W. (2023). Benefits of P-Coumaric Acid in Mice with Rotenone-Induced Neurodegeneration. Metab. Brain Dis..

[40] Bazyar H., Zare Javid A., Ahangarpour A., Zaman F., Hosseini S.A., Zohoori V., Aghamohammadi V., Yazdanfar S., Ghasemi Deh Cheshmeh M. (2023). The Effects of Rutin Supplement on Blood Pressure Markers, Some Serum Antioxidant Enzymes, and Quality of Life in Patients with Type 2 Diabetes Mellitus Compared with Placebo. Front. Nutr..

[41] Kaur J., Kaur R. (2022). P-Coumaric Acid: A Naturally Occurring Chemical with Potential Therapeutic Applications. Curr. Org. Chem..

[42] Espíndola K.M.M., Ferreira R.G., Narvaez L.E.M., Silva Rosario A.C.R., da Silva A.H.M., Silva A.G.B., Vieira A.P.O., Monteiro M.C. (2019). Chemical and Pharmacological Aspects of Caffeic Acid and Its Activity in Hepatocarcinoma. Front. Oncol..

[43] Gupta N., Verma K., Nalla S., Kulshreshtha A., Lall R., Prasad S. (2020). Free Radicals as a Double-Edged Sword: The Cancer Preventive and Therapeutic Roles of Curcumin. Molecules.

[44] Bhosale P.B., Ha S.E., Vetrivel P., Kim H.H., Kim S.M., Kim G.S. (2020). Functions of Polyphenols and Its Anticancer Properties in Biomedical Research: A Narrative Review. Transl. Cancer Res..

[45] Farghadani R., Naidu R. (2023). The Anticancer Mechanism of Action of Selected Polyphenols in Triple-Negative Breast Cancer (TNBC). Biomed. Pharmacother..

[46] Niedzwiecki A., Roomi M.W., Kalinovsky T., Rath M. (2016). Anticancer Efficacy of Polyphenols and Their Combinations. Nutrients.

[47] Kotsou K., Magopoulou D., Chatzimitakos T., Athanasiadis V., Bozinou E., Sfougaris A.I., Lalas S.I. (2024). Enhancing the Nutritional Profile of *Crataegus monogyna* Fruits by Optimizing the Extraction Conditions. Horticulturae.

[48] Chatzimitakos T., Athanasiadis V., Makrygiannis I., Kalompatsios D., Bozinou E., Lalas S.I. (2023). An Investigation into *Crithmum maritimum* L. Leaves as a Source of Antioxidant Polyphenols. Compounds.

[49] Chatzimitakos T., Athanasiadis V., Kotsou K., Mantiniotou M., Kalompatsios D., Makrygiannis I., Bozinou E., Lalas S.I. (2024). Optimization of Pressurized Liquid Extraction (PLE) Parameters for Extraction of Bioactive Compounds from *Moringa oleifera* Leaves and Bioactivity Assessment. Int. J. Mol. Sci..

