# Supplementary material for: Exploring Conventional and Green Extraction Methods for Enhancing the Polyphenol Yield and Antioxidant Activity of Hyssopus officinalis Extracts
Source: Plants (Basel). 2024 Jul 29;13(15):2105. doi: 10.3390/plants13152105 (PMC11313759; doi:10.3390/plants13152105)
Supplement: Supplementary file 1 [file plants-13-02105-s001.zip › plants-3060481-supplementary.pdf]

Supplementary material

Exploring Conventional and Green Extraction Methods for Enhancing Polyphenol yield and Antioxidant Activity of *Hyssopus officinalis* Extracts

Sofia Polaki, Vasiliki Stamatelopoulou, Konstantina Kotsou, Theodoros Chatzimitakos *, Vassilis Athanasiadis, Eleni Bozinou and Stavros I. Lalas

Department of Food Science & Nutrition, University of Thessaly, Terma N. Temponera Str., 43100 Karditsa, Greece; spolaki@uth.gr (S.P.); vstamatel@uth.gr (V.S.); kkotsou@agr.uth.gr (K.K.); vaathanasiadis@uth.gr (V.A.); empozinou@uth.gr (E.B.); slalas@uth.gr (S.I.L.)

* Correspondence: tchatzimitakos@uth.gr

3.1. Chemicals and Reagents

All solvents were at least of HPLC grade and purchased from Carlo Erba (Val de Reuil, France). Chemical standards of polyphenolic compounds, such as, neochlorogenic, catechin, rutin, quercetin 3-*D*-galactoside, luteolin, apigenin, apigenin-7-*O*-glucoside, chlorogenic acid, vanillic acid, caffeic acid, syringic acid, *p*-coumaric acid, ferulic acid were acquired from Sigma-Aldrich (Steinheim, Germany). Hydrochloric acid, ascorbic acid, trichloroacetic acid, ferric (III) chloride, aluminum chloride, and sodium acetate were also obtained from Sigma-Aldrich (Steinheim, Germany). Gallic acid, anhydrous sodium carbonate, Folin-Ciocalteu reagent, 2,2-diphenyl-1-picrylhydrazyl (DPPH), and 2,4,6-tri-2-pyridinyl-1,3,5-triazine (TPTZ) were obtained by Penta (Prague, Czech Republic). For all experiments, deionized water was used.

3.4. Response Surface Methodology (RSM) Optimization of Extraction and Experiment Design

Utilizing the Response Surface Methodology (RSM) technique, the extraction of total polyphenol content (TPC), polyphenolic compounds, and antioxidant activity was measured using the FRAP, DPPH method aimed for optimal yield. The design's objective was to enhance the content of *H. officinalis* extracts in TPC, polyphenolic compounds, and antioxidant activity. This was achieved through adjustments to the extraction procedure involving parameters such as solvent concentration (ethanol, EtOH) represented as *C*, % *v*/*v* (chosen to investigate different polarity solvents), extraction duration denoted as *t*, min (selection was based on preliminary experiments), and extraction temperature indicated as *T*, °C (as ethanol has a boiling point of 78.3 °C, a maximum temperature of 80 °C was chosen to ensure the stability of the extracted compounds and the feasibility of the extraction process). An experiment employing a Main Effect Screening design with twenty design points formed the basis for optimization. Process variables were set at five levels, as outlined in Table 7, indicating both coded and actual levels. Analysis of variance (ANOVA) and summary-of-fit tests were employed to establish overall model significance (R^2, *p*-value) and the significance of model coefficients (equations). Additionally, a second-order polynomial model (Equation S1) was utilized to forecast the dependent variable based on the analyzed independent factors:

$$Y_k = \beta_0 + \sum_{i=1}^{2} \beta_i X_i + \sum_{i=1}^{2} \beta_{ii} X_i^2 + \sum_{i=1}^{2} \sum_{j=i+1}^{3} \beta_{ij} X_i X_j \quad \text{(S1)}$$

where Y_k is the predicted response variable; X_i and X_j are the independent variables; β_0, β_i, β_{ii}, and β_{ij} are the intercept, regression coefficients of the linear, quadratic, and interaction terms of the model, respectively.

A

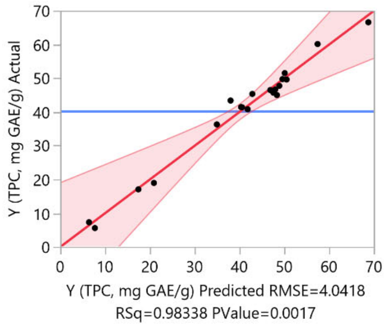

Summary of Fit

RSquare	0.9834
RSquare Adj	0.9368
Root Mean Square Error	4.0418
Mean of Response	40.361
Observations (or Sum Wgts)	20

Analysis of Variance

Source	DF	Sum of Squares	Mean Square	F Ratio
Model	14	4832.49	345.18	21.129
Error	5	81.6823	16.336	Prob > F
C. Total	19	4914.17		0.0017*

Parameter Estimates

Term	Estimate	Std Error	t Ratio	Prob>|t|
Intercept	41.324	3.3519	12.33	<0.0001*
X1(1,4)	-5.168	1.432	-3.61	0.0154*
X2(1,5)	-20.54	2.3891	-8.60	0.0004*
X3(1,5)	4.0993	3.4574	1.19	0.2890
X4(1,5)	1.7974	2.2586	0.80	0.4622
X1*X1	1.8046	2.5544	0.71	0.5115
X1*X2	6.5937	2.5096	2.63	0.0467*
X2*X2	-8.71	5.8087	-1.50	0.1940
X1*X3	-9.944	4.6993	-2.12	0.0879
X2*X3	-7.345	5.3472	-1.37	0.2280
X3*X3	5.6267	2.8437	1.98	0.1048
X1*X4	5.2777	3.4952	1.51	0.1914
X2*X4	8.8012	5.7295	1.54	0.1851
X3*X4	-6.665	3.291	-2.03	0.0987
X4*X4	0.2757	4.299	0.06	0.9513

B

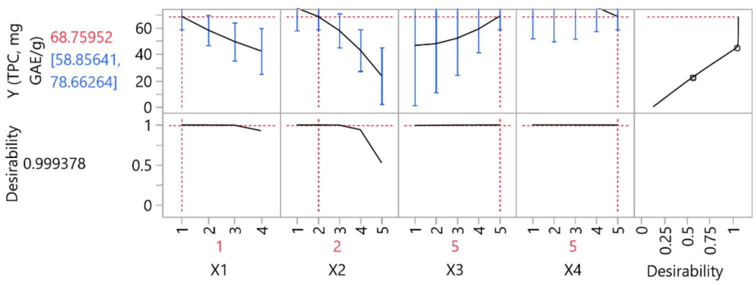

Figure S1. Plot **A** displays the actual response versus the predicted response (Total polyphenol content – TPC, mg GAE/g) for optimizing *H. officinalis* extracts using hydroethanolic solutions, different extraction techniques, and parameters, and plot **B** displays the desirability function. Asterisks and colored values denote statistically significant values, while inset tables include statistics relevant to evaluating the resulting model.

A

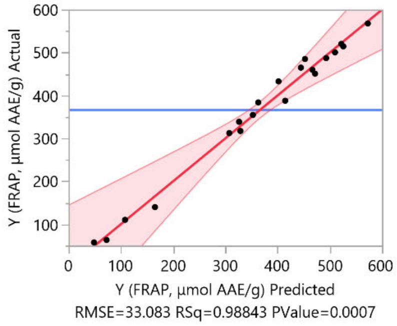

Summary of Fit

RSquare	0.9884
RSquare Adj	0.956
Root Mean Square Error	33.083
Mean of Response	367.37
Observations (or Sum Wgts)	20

Analysis of Variance

Source	DF	Sum of Squares	Mean Square	F Ratio
Model	14	467474	33391	30.508
Error	5	5472.4	1094.5	Prob > F
C. Total	19	472946		0.0007*

Parameter Estimates

Term	Estimate	Std Error	t Ratio	Prob>|t|
Intercept	437.8	27.436	15.96	<0.0001*
X1(1,4)	-43.44	11.721	-3.71	0.0139*
X2(1,5)	-172.4	19.555	-8.82	0.0003*
X3(1,5)	7.3419	28.299	0.26	0.8056
X4(1,5)	14.022	18.487	0.76	0.4824
X1*X1	-67.86	20.909	-3.25	0.0228*
X1*X2	78.383	20.542	3.82	0.0124*
X2*X2	-163.3	47.545	-3.43	0.0185*
X1*X3	-39.04	38.464	-1.01	0.3567
X2*X3	-55.47	43.768	-1.27	0.2608
X3*X3	66.835	23.276	2.87	0.0349*
X1*X4	56.192	28.609	1.96	0.1067
X2*X4	46.949	46.896	1.00	0.3627
X3*X4	-41.12	26.938	-1.53	0.1874
X4*X4	40.286	35.188	1.14	0.3041

B

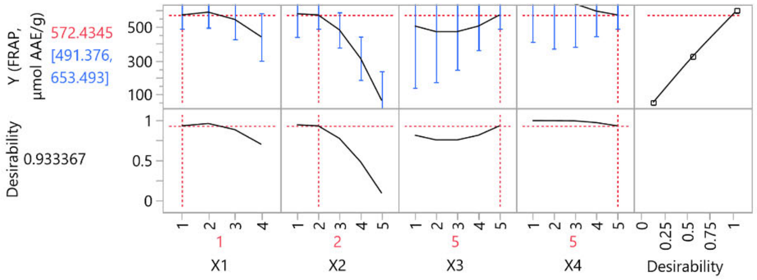

Figure S2. Plot **A** displays the actual response versus the predicted response (FRAP, µmol AAE/g) for optimizing *H. officinalis* extracts using hydroethanolic solutions, different extraction techniques, and parameters, and plot **B** displays the desirability function. Asterisks and colored values denote statistically significant values, while inset tables include statistics relevant to evaluating the resulting model.

A

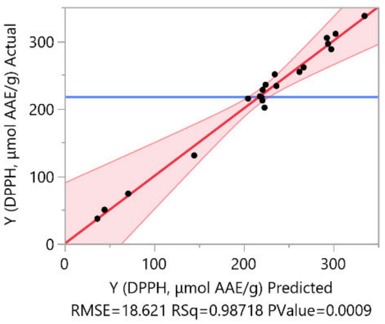

Summary of Fit

RSquare	0.9872
RSquare Adj	0.9513
Root Mean Square Error	18.621
Mean of Response	217.56
Observations (or Sum Wgts)	20

Analysis of Variance

Source	DF	Sum of Squares	Mean Square	F Ratio
Model	14	133549	9539.2	27.511
Error	5	1733.7	346.74	**Prob > F**
C. Total	19	135283		0.0009*

Parameter Estimates

Term	Estimate	Std Error	t Ratio	Prob>|t|
Intercept	177.64	15.443	11.50	<0.0001*
X1(1,4)	5.3739	6.5972	0.81	0.4524
X2(1,5)	-116.2	11.007	-10.56	0.0001*
X3(1,5)	27.018	15.928	1.70	0.1506
X4(1,5)	11.937	10.405	1.15	0.3032
X1*X1	16.326	11.768	1.39	0.2240
X1*X2	7.1623	11.562	0.62	0.5627
X2*X2	26.698	26.761	1.00	0.3643
X1*X3	-118.9	21.65	-5.49	0.0027*
X2*X3	-33.97	24.635	-1.38	0.2265
X3*X3	53.528	13.101	4.09	0.0095*
X1*X4	52.029	16.103	3.23	0.0232*
X2*X4	79.728	26.396	3.02	0.0294*
X3*X4	-43.26	15.162	-2.85	0.0357*
X4*X4	-15.33	19.806	-0.77	0.4740

B

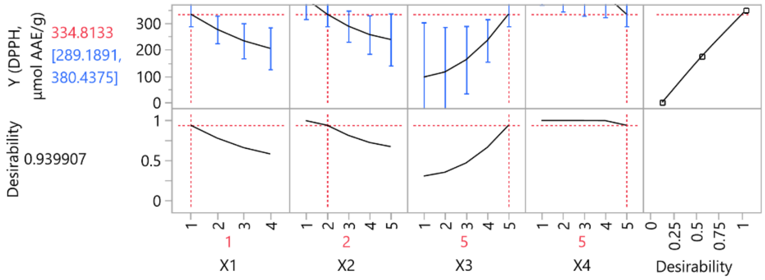

Figure S3. Plot **A** displays the actual response versus the predicted response (DPPH, µmol AAE/g) for optimizing *H. officinalis* extracts using hydroethanolic solutions, different extraction techniques, and parameters, and plot **B** displays the desirability function. Asterisks and colored values denote statistically significant values, while inset tables include statistics relevant to evaluating the resulting model.

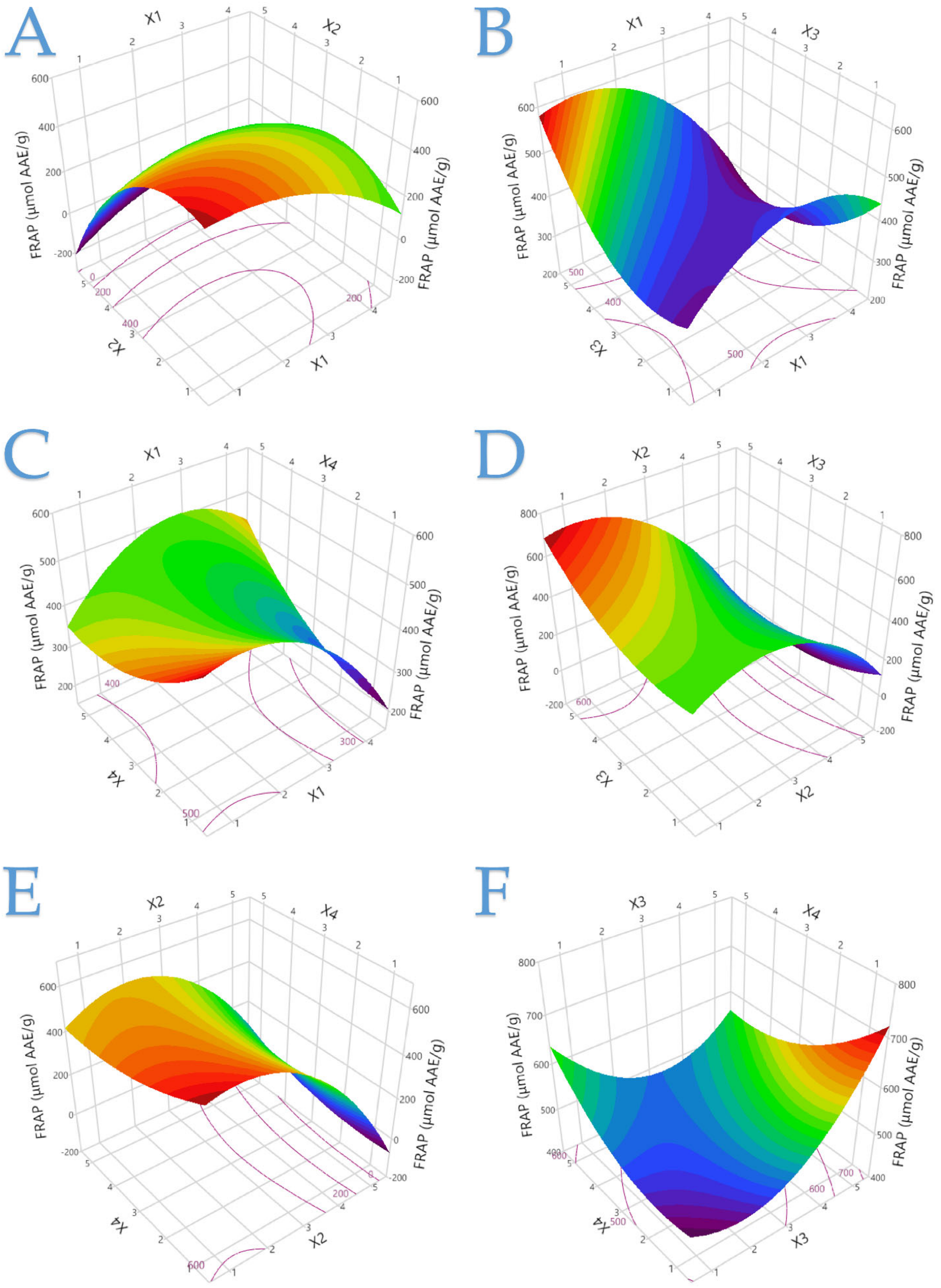

Figure S4. The optimal extraction of the *H. officinalis* plant is shown in 3D graphs that show the impact of the process variables considered in the response (FRAP, µmol AAE/g). Plot (**A**), covariation of X_1 and X_2; plot (**B**), covariation of X_1 and X_3; plot (**C**), covariation of X_1 and X_4; plot (**D**), covariation of X_2 and X_3; plot (**E**), covariation of X_2 and X_4; plot (**F**), covariation of X_3 and X_4.

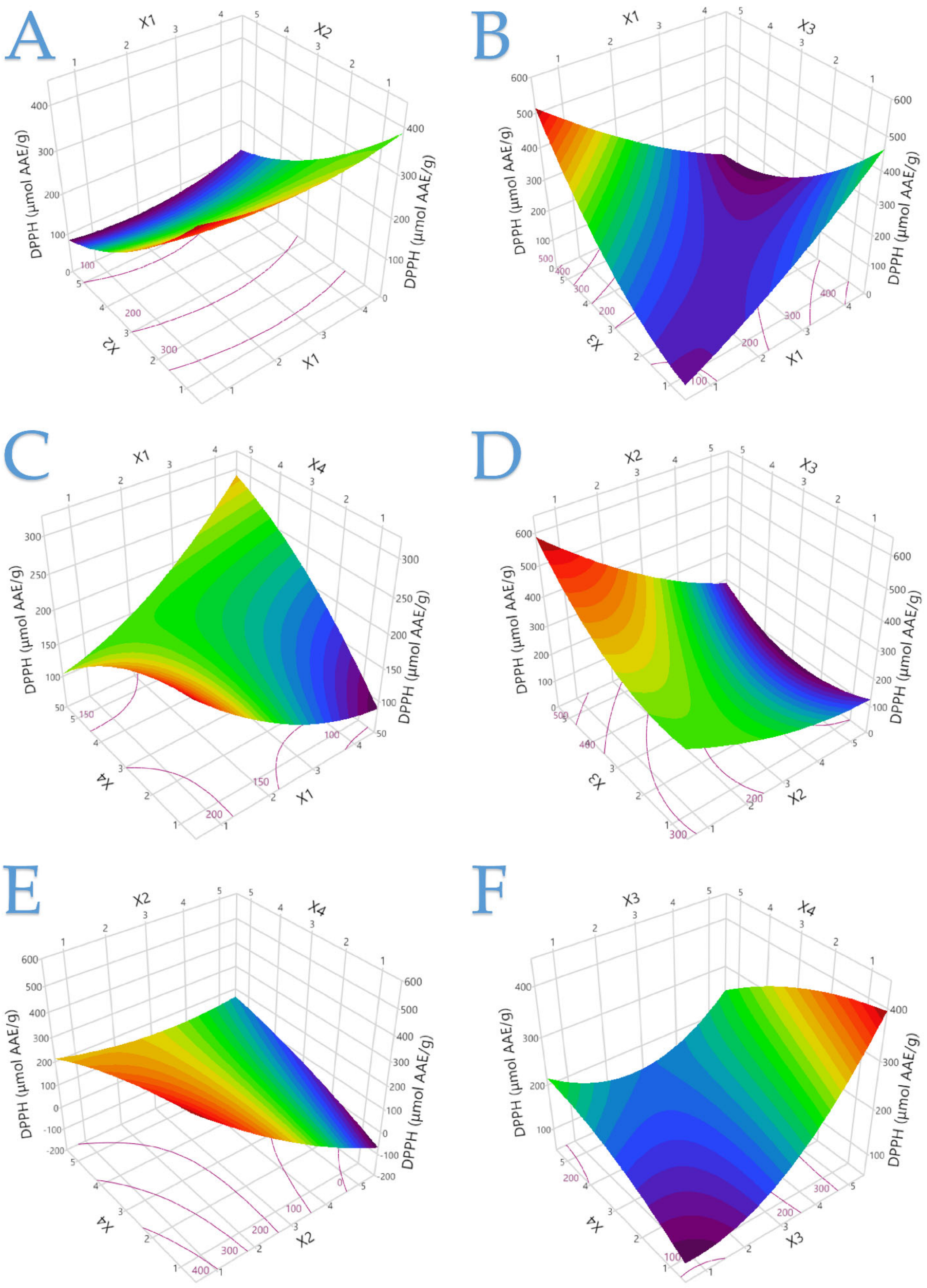

Figure S5. The optimal extraction of the *H. officinalis* plant is shown in 3D graphs that show the impact of the process variables considered in the response (DPPH, µmol AAE/g). Plot (**A**), covariation of X_1 and X_2; plot (**B**), covariation of X_1 and X_3; plot (**C**), covariation of X_1 and X_4; plot (**D**), covariation of X_2 and X_3; plot (**E**), covariation of X_2 and X_4; plot (**F**), covariation of X_3 and X_4.

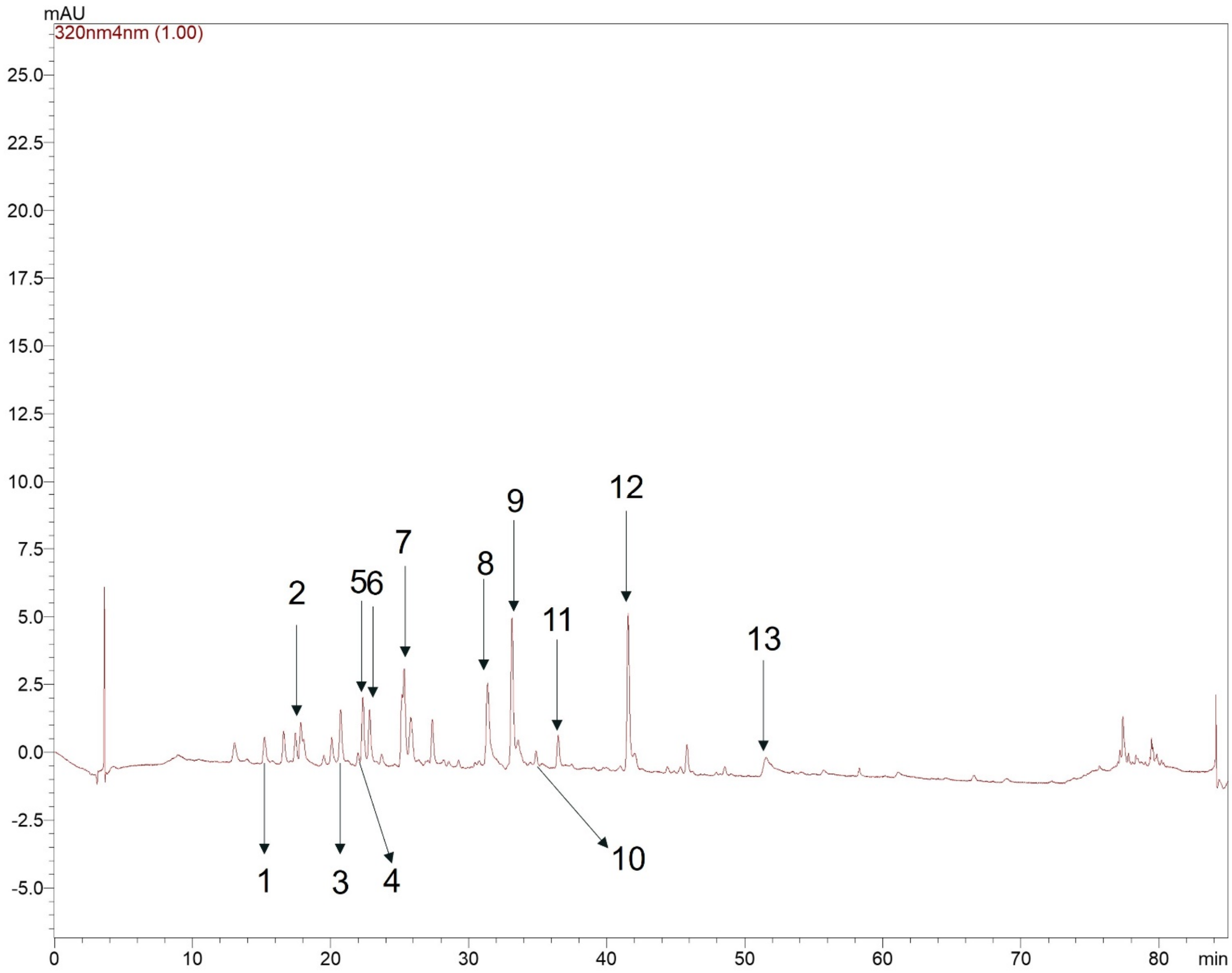

Figure S6. Representative HPLC chromatogram at 320 nm of the optimal extract of *H. officinalis* plant demonstrating the identified compounds. 1: Neochlorogenic acid; 2: Catechin; 3: Chlorogenic acid; 4: Vanillic acid; 5: Caffeic acid; 6: Syringic acid; 7: *p*-Coumaric acid; 8: Ferulic acid; 9: Rutin; 10: Quercetin 3-*D*-galactoside; 11: Luteolin-7-glucoside; 12: Apigenin-7-*O*-glucoside; 13: Apigenin.

Table S1. Data for the identification and quantification of polyphenolic compounds in the extracts of *H. officinalis* plant.

Polyphenolic Compound	Retention Time (min)	Absorbance Maximum (nm)	Equation	R^2
Neochlorogenic acid	15.2	324	y = 28,213.51x + 551.72	0.9987
Catechin	17.8	278	y = 11,920.79x − 128.19	0.9973
Chlorogenic acid	20.7	325	y = 50,320.40x − 23,038.36	0.9943
Vanillic acid	21.9	270	y = 20,000x + 1224	0.9939
Caffeic acid	22.3	322	y = 937,658.95x + 12,216.24	0.9998
Syringic acid	22.8	270	y = 20,000x + 1687	0.9985
p-Coumaric acid	25.3	309	y = 120,568.59x + 1059.043	0.9998
Ferulic acid	31.3	322	y = 108,553.73x - 25,916.43	0.9992
Rutin	33.1	254	y = 46,365.62x - 31,562.74	0.9970
Quercetin 3-*D*-galactoside	34.8	257	y = 41,489.69x − 35,577.55	0.9934
Luteolin-7-glucoside	36.4	347	y = 34,875.94x − 16,827.36	0.9993
Apigenin-7-*O*-glucoside	41.5	336	y = 64,742.65x + 15,897.94	0.9979
Apigenin	51.5	227	y = 96,840x − 63,647	0.9975
